# Checking Perspiration

**Published:** 1877-08

**Authors:** 


### CHECKING PERSPIRATION.

A Boston merchant, in "lending a hand" on board of one of his ships on a windy day, found himself at the end of an hour and a half pretty well exhausted and perspiring freely. He sat down to rest. The cool wind from the sea was delightful, and engaging in conversation, time passed faster than he was aware of. In attempting to rise, he found he was unable to do so without assistance. He was taken home and put to bed, where he remained two years; and for a long time afterwards, could only hobble about with the aid of a crutch. Less exposures than this have, in constitutions not so vigorous, resulted in inflammation of the lungs, "pneumonia," ending in death in less than a week, or causing tedious rheumatisms, to be a source of torture for a lifetime. Multitudes of lives would be saved every year, and an incalculable amount of human suffering would be prevented, if parents would begin to explain to their children at the age of three or four years, the danger which attends cooling off too quickly after exercise, and the importance of not standing still after exercise, or work, or play, or of remaining exposed to a wind, or of sitting at open window or door, or of pulling off any garment, even the hat or bonnet, while in a heat. It should be remembered by all, that a cold never comes without a cause, and that in four times out of five, it is the result of leaving off exercise too suddenly or of remaining still in the wind, or in a cooler atmosphere than that in which the exercise has been taken.

The colder the weather the more need is there, in coming into the house, to keep on all the clothing, except India-rubber or damp shoes, for several minutes afterwards. Very few rooms are heated higher than sixty-five degrees when the thermometer is within twenty degrees of zero, while the temperature of the body is always at ninety-eight, in health; so that if a man comes into a room which is thirty degrees colder than his body, he will rapidly cool off, too much so often, even if the external clothing is not removed.

It is not necessary that the perspiration be visible; any exercise which excites the circulation beyond what is natural, causes a proportional increase of perspiration, the sudden checking of which induces dangerous diseases and certain death every day.

